# Using The Cancer Genome Atlas as an Inquiry Tool in the Undergraduate Classroom

**DOI:** 10.3389/fgene.2020.573992

**Published:** 2020-12-16

**Authors:** William Hankey, Nicholas Zanghi, Mackenzie M. Crow, Whitney H. Dow, Austin Kratz, Ashley M. Robinson, Meaghan R. Robinson, Verónica A. Segarra

**Affiliations:** ^1^Department of Pathology, Duke Cancer Center, Duke University, Durham, NC, United States; ^2^Department of Biology, High Point University, High Point, NC, United States; ^3^Department of Chemistry, High Point University, High Point, NC, United States; ^4^Department of Physics, High Point University, High Point, NC, United States

**Keywords:** bioinformatics, cancer, genomics, cancer genomics, undergraduate teaching and learning

## Abstract

Undergraduate students in the biomedical sciences are often interested in future health-focused careers. This presents opportunities for instructors in genetics, molecular biology, and cancer biology to capture their attention using lab experiences built around clinically relevant data. As biomedical science in general becomes increasingly dependent on high-throughput data, well-established scientific databases such as The Cancer Genome Atlas (TCGA) have become publicly available tools for medically relevant inquiry. The best feature of this database is that it bridges the molecular features of cancer to human clinical outcomes—allowing students to see a direct connection between the molecular sciences and their future professions. We have developed and tested a learning module that leverages the power of TCGA datasets to engage students to use the data to generate and test hypotheses and to apply statistical tests to evaluate significance.

## Introduction

While many undergraduates are interested in becoming medical doctors and declare “pre-med” early in their academic careers, it is predicted that by 2032 the United States will face a shortage of between 46,900 and 121,900 physicians (Dall et al., 2019). One of the factors likely to exacerbate this projected shortage is the high attrition rates of undergraduates from the premedical academic track (Lin et al., 2013). In fact, many of the empirical studies recorded in the scientific literature related to undergraduate premedical students are focused on documenting and better understanding attrition from the premedical track (Lin et al., 2013). High attrition rates in undergraduate premedical tracks have been found to be influenced by a variety of factors including loss of interest and negative experiences in required courses (Lin et al., 2013).

Student interest and persistence in STEM careers can be increased and strengthened through participation in Course-based Undergraduate Research Experiences (CUREs) as part of the curriculum (Estrada et al., 2016). These findings suggest that one of the ways in which student persistence in undergraduate premedical programs can be increased is through relevant CURE experiences that highlight clinically relevant data and its applications.

While undergraduate access to clinical research experiences is limited, the biomedical sciences are becoming increasingly dependent on high-throughput data, and well-established scientific databases such as The Cancer Genome Atlas (TCGA) have become publicly available tools for medically relevant inquiry (Cancer Genome Atlas Network, 2012; Cerami et al., 2012; Gao et al., 2013). These databases are increasingly being recognized as resources available for undergraduate teaching (Coughlan, 2020).

Furthermore, there is currently a need for physicians and health professionals to recognize and use the power of cutting edge genomics to inform diagnosis and treatments for their patients (Rubanovich et al., 2018). Through the use of clinically relevant genomic datasets like the ones found in TCGA in the undergraduate classroom, we can raise awareness for the relevance of these resources in medicine early on in the training of these individuals (Schoenborn et al., 2019).

It is also important to point out the increasing need for scientific literacy, pro-science attitudes, and evidence-based decision-making among non-majors in a variety of different disciplines (Ballen et al., 2017). These skills, including scientific literacy, can be developed using CURE experiences and inquiry-based modules in the non-majors classroom (Ballen et al., 2017; Segarra et al., 2018).

We have developed and tested a learning module that leverages the power of TCGA datasets to engage students in inquiry-based clinical research in the context of cancer—a human disease that is of universal relevance. Our module allows students to not only generate and test hypotheses with clinical relevance, but also apply statistical tests to evaluate significance. Continuing to refine such activities to better cultivate engagement in and comfort with data-based decision-making will better position us to foster interest, persistence, and scientific literacy among undergraduate science majors both inside and outside of the premedical track, as well as non-majors preparing to enter an increasingly data-driven workplace.

## Methodology

### Accessing TCGA Datasets

The Cancer Genome Atlas data were accessed by the course instructor through cBioportal^1^, a widely used web interface that provides access to public cancer genomics datasets (Cancer Genome Atlas Network, 2012; Cerami et al., 2012; Gao et al., 2013). Breast cancer was selected as a focus because of the increased likelihood for the intended audience members to make personal connections to a highly prevalent cancer type with a significant impact on human health, and because of the convenience of introducing the genomic data starting with familiar genes such as *BRCA1*, *BRCA2*, and the gene encoding p53 (*TP53*) that had previously been discussed in the lecture component of the class. TCGA was chosen as a data source for the combination of high-quality genomic and associated clinical data characteristic of TCGA datasets in general and the high sample size of the available datasets. The TCGA Breast Invasive Carcinoma dataset associated with the 2015 publication in *Cell* (Ciriello et al., 2015) was specifically chosen from among the four available TCGA Breast Invasive Carcinoma datasets because of its combination of mutation data and copy number alteration data, as well as its inclusion of stage among the clinical data variables (study ID “brca_tcga_pub2015”; https://www.cbioportal.org/study/summary?id==brca_tcga_pub2015). It should be noted that the original data set was composed of a total of 818 patient samples—817 from primary and 1 from metastatic tumors. Only data from the 817 primary tumor samples were included in the student analysis. The metastatic sample was excluded in order to present the students with a comparable and consistent group of samples for analysis.

While mutation and copy number data were available in the dataset for more than 20,000 genes, a more focused subset of 16 total genes was selected to provide to the students. This subset was narrow enough facilitate visualization of the complete dataset and analysis by first-time bioinformaticists in Microsoft Excel, but diverse enough to include examples fitting several different patterns. The list began with well-known cancer-associated genes previously discussed in the course (*BRCA1*, *BRCA2*, *TP53*), then added genes that were among the most frequently targeted in breast cancer by known pathogenic mutations (*PIK3CA*, *CDH1*, *GATA3*, *MAP3K1*, *KMT2C*, and *AKT1*), amplifications (*MYC*, *CCND1*, and *ERBB2*), or deletions (*RB1*, *PTEN*). These high-frequency targets of mutations and copy number alterations were identified by selecting the dataset of interest (Cancer Genome Atlas Network, 2012) from the cBioportal menu and using the Explore Selected Studies function to view the Summary of findings. The genes encoding β-actin (*ACTB*) and hemoglobin subunit β (*HBB*) were added to the list in order to function as recognizable negative control genes generally not associated with cancer. Once the list of sixteen breast cancer-relevant genes and controls was determined, the 16 gene names were entered as a list into the cBioportal website to access genomics data for this subset using the Query by Gene function. For each gene of interest, genetic mutation data and copy number alteration data were separately accessed for all 817 tumors in the dataset from the Download section of the site, selecting the Tab Delimited Format option. Clinical data were accessed through the cBioportal site using the Explore Selected Studies function and the Clinical Data tab. A limited subset of clinical characteristics were downloaded, with each characteristic chosen to help illustrate a different point or to enable the students to test a different hypothesis. The majority of clinical variables were categorical, facilitating the use of 2 × 2 tables to test association between the clinical category and the status of a gene as mutated/unmutated, etc. The 15 characteristics were Informed Consent by Patient (Yes/No), Diagnosis Age, Cancer Type, Race Category, Ethnicity Category, Sex, Disease Stage (I–IV), Treatment Outcome (Living Disease-Free/Living with Tumor/Recurred, or Progressed/Deceased), Time from Treatment to Recurrence (Months), Time from Treatment to Death (Months), Time from Treatment to Most Recent Contact (Months), ER Status (by Staining), PR Status (by Staining), HER2 Status (by Staining), and Total Number of Mutations. Similar to the mutation and copy number data files, the clinical data were arrayed so that the clinical variables were each assigned a different column, while the 817 tumors were each assigned a different row (Supplementary Appendix 1).

### Combining Genetic and Clinical TCGA Data in Microsoft Excel

Initially, the separate Mutations and Copy-Number Alterations files were integrated into a single Excel file by alphabetizing the list of samples in each file by Patient ID and integrating the columns along matching rows. The instructor then sought to integrate the mutation status and the copy number status into a single column for each gene, stating only the change in that gene most relevant to the disease. For example, if the Copy Number Alteration column for *TP53* listed the gene as Amplified in a particular tumor, while the Mutations column for *TP53* listed it as a known Pathogenic Mutant in that same tumor, the merging of those two columns into one *TP53* Status column listed it as Pathogenic Mutant for that tumor. On the other hand, if the Copy Number Alteration column for *TP53* listed the gene as Amplified in a particular tumor, while the Mutations column for *TP53* listed it as a Mutant of Unknown Significance in that same tumor, the merging of those two columns into one *TP53* Status column listed it as Amplified for that tumor. The resulting Excel file containing gene status data was then integrated with the Clinical Data file into a single Excel file by alphabetizing the list samples in each file by Patient ID and integrating them along matching rows. The resulting file contained 16 columns of genetic data and 15 columns of clinical data, with 817 rows of tumor samples, each representing a different patient (Supplementary Appendix 1).

### Generation of the Worksheet

The instructor designed an assignment to introduce students to the kinds of research hypotheses that are testable using the combination of genetic and clinical data. The initial assignment was generated in the form of a worksheet (Supplementary Appendix 2), which consisted of five different tables. Categorical clinical and/or genetic characteristics were listed along the *x*- and *y*-axes, and students were asked to count how many tumors from the dataset possessed each combination of characteristics. Students first determined how many of the patients classified as Living Disease-Free, Living with Tumor, Recurred or Progressed, and Deceased were diagnosed with Stage I vs. II vs. III vs. IV tumors. This comparison of stage and outcome was selected to illustrate a well-known clinical association and presented students with an opportunity to test whether the counts matched their expectations. Students then determined how many of the patients classified as Living Disease-Free, Living with Tumor, Recurred or Progressed, and Deceased harbored vs. did not harbor pathogenic mutations/deletions in *TP53*, *BRCA1*, or *BRCA2*. Students were already familiar with all three genes as well-known tumor suppressors in breast cancer, and were able to formulate hypotheses about how mutations in each gene might associate with clinical outcome. In the final table, students were asked to calculate the total number of tumors with pathogenic mutant, mutant of unknown significance, amplified, and deleted genotypes, for each of the sixteen genes. Since most of these genes were less familiar, students would have the opportunity to collect the data without bias, and then to use them to form a hypothesis about each gene’s status as an oncogene, tumor suppressor gene, or neither.

### Generation of Instructions for Sorting Tumors in Microsoft Excel

Students came into the assignment with heterogeneous backgrounds using Microsoft Excel for similar tasks, and were provided with general instructions to help them complete the worksheet (Supplementary Appendix 3). The Sort and Filter function in Excel was recommended as a critical tool for organizing data into subsets according to a particular genomic or clinical characteristic. Within each subset, students were recommended to count occurrences of the associated characteristic using the COUNTIF function in combination with quotation marks around the text of interest.

### Generation of a Microsoft Excel File to Support Statistical Analysis

As a follow-up assignment, students were asked to use the counts data from their completed worksheet to generate one hypothesis about the association of two variables. They would then construct a 2 × 2 table and perform a test for statistically significant association. The chi-square test of independence was recommended as an applicable statistical test that can be performed using Excel. To facilitate their introduction to this statistical test, a template Excel file was constructed into which the students could enter their 2 × 2 table (Supplementary Appendix 4). The file would then use these observed counts to calculate the expected counts, determine the test statistic, and generate a *p*-value.

## Classroom Implementation

The documents/data described above (also see Supplementary Materials) were used to create and implement a bioinformatics laboratory experience during two 3-h lab periods near the conclusion of an upper-level undergraduate Cancer Biology course. This activity can also be implemented in a bioinformatics or genetics course and is particularly well suited to be implemented remotely in the context of online teaching.

**Step 1:** *Introduce students to the Microsoft Excel file containing data subset of interest.*

Students were introduced to the data subset of interest, including the kind of information each column and row contained (Supplementary Appendix 1).

**Step 2:** *Students complete a worksheet composed of 2* × *2 tables that measure associations between presence/absence of a mutation and categorical clinical phenotypes.*

Students were given the opportunity to increase their familiarity with the dataset of interest (Supplementary Appendix 1) by completing an Excel worksheet (Supplementary Appendix 2) that required them to identify the data relevant to different categories. To help students sift through the data, they were provided with tips for sorting tumor data in Excel (Supplementary Appendix 3).

**Step 3:** *Students articulate a new association of interest to test (research question), create/complete the appropriate 2* × *2 tables, and calculate statistical significance of association.*

Using the data as a guide, students were given the opportunity to come up with their own association or research question to test (Table 1). Students had to examine the data provided and decide which two categorical variables they wanted to use to test an association. Students were introduced to the chi-square test of independence and its relevance to categorical data. To facilitate students performing the relevant statistics, an Excel file template was provided (Supplementary Appendix 4). Before beginning this portion of the assignment, the instructor demonstrated the process from selection of an association of interest and 2 × 2 table construction, all the way to statistical analysis.

**TABLE 1:** Representative research questions answered by students using TCGA Breast Invasive Carcinoma datasets.

Research question	Categorical variables being compared	*p*-value
Are pathogenic *PIK3CA* gene mutations associated with poor clinical outcomes (not living disease-free) for breast cancer?	Wildtype *PIK3CA* vs. pathogenic mutations in *PIK3CA* Good clinical outcome (living disease-free) vs. Poor clinical outcome (not living disease-free)	0.24
Is the wildtype *BRCA2* gene associated with good (living disease-free) breast cancer clinical outcomes?	Wildtype *BRCA2* vs. pathogenic mutant *BRCA2* Good clinical outcome (living disease-free) vs. Poor clinical outcome (not living disease-free)	0.93
Are *BRCA1* gene tumor mutations associated with poor (not living disease-free) breast cancer outcomes?	Wildtype *BRCA1* vs. mutated BRCA1 gene Good clinical outcome (living disease-free) vs. Poor clinical outcome (not living disease-free)	0.60
Are pathogenic *TP53* mutations associated with more advanced (Stages II/III/IV) stages of cancer?	Wildtype *TP53* vs. pathogenic *TP53* gene mutations Early (Stage I) vs. advanced stages of cancer (Stages II/III/IV)	0.14
Are pathogenic *BRCA1* mutations associated with breast cancer recurrence?	Wildtype *BRCA1* vs. pathogenic *BRCA1* mutants Good clinical outcome (living disease-free) vs. Poor clinical outcome (living but tumor recurred/progressed)	0.01
Are patients living disease-free more likely to have been diagnosed early stage breast cancer (Stages I/II)?	Living disease-free vs. Not living disease free Early stage (Stages I/II) vs. late stage (III/IV) cancer	2 × 10^–6^

*For Step 3 in Classroom Implementation, students articulate a new association of interest to test (research question), create/complete 2 × 2 tables, and calculate its statistical significance. Shown in this table are representative research questions (associations being tested), including the categorical variables being tested and the determined statistical significance (*p*-value) of the association. Associations that were not independent from each other have a *p*-value less or equal than 0.05.*

Microsoft Excel was selected for this activity due to its familiarity to the majority of undergraduate students as both a calculator and a tool for generating scientific figures. Thus, it serves as a comfortable starting point in which the dimensions of the dataset can be visualized and new functions and calculations for data analysis can be introduced. At the same time, it is important to note the caveat that Microsoft Excel is increasingly recognized as a flawed platform for statistical analysis. In comparison to the open-source programming language R, which has become a preferred platform for many research applications of statistics, Excel is considered the less reproducible and more error-prone option (Ziemann et al., 2016). A key advantage of R is the ability to record and share in a transparent way the steps taken to organize and analyze the data (Incerti et al., 2019). While we felt that the benefits of Excel outweighed the caveats in this particular application, future adaptations of this exercise might consider introducing students instead to programming in R or to other commercial software packages for statistics and data science, such as Stata, SPSS, SAS, or JMP. Substitution of these tools for Excel might create an additional obstacle to the accessibility of key concepts to students, but would likely benefit those students who might continue to use these programs in their future research.

## Discussion

While, at first, students had difficulty managing the large amount of information that was provided, sharing strategies to sort and count data using Excel helped them gain confidence in using the dataset to complete Steps 2 and 3 described above. In fact, all students were ultimately able to get perfect grades on their practice worksheets (Supplementary Appendix 2).

Table 1 provides representative research questions that were answered using the breast cancer tumor data available. In general, many of the associations tested were not statistically significant. This is likely due to shortcomings of the dataset that have been noted and described by others (Huo et al., 2017; Liu et al., 2018). For example, clinical annotation of TCGA datasets with patient survival and treatment outcomes is incomplete—follow-up times are short (TCGA only stayed in touch with clinicians regarding their patients’ clinical outcomes for a short period of time) and data is unclear at times about what the cause of death actually was (may not have been cancer; Huo et al., 2017). Moreover, breast cancer is a less aggressive cancer type, and can take 10 years or more to recur (Liu et al., 2018). So given the relatively short window of follow-up time during which TCGA outcomes were measured (reported by clinicians following up on their patients), overall survival is not a suitable clinical outcome to use (Liu et al., 2018). Overall survival is also complicated by other causes of death besides breast cancer. Disease-free survival/recurrence might have been a better endpoint to use (Liu et al., 2018). While these factors may compromise the accuracy of correlations to survival and staging, they do not affect the primary goal of using these data as a tool for learning in the classroom.

Table 2 provides student feedback that captures their attitudes and perceptions about the TCGA modules described in this paper. While students reported being initially taken aback by the size of the dataset, they reported that completing the worksheet and learning new Excel commands like the COUNTIF function helped them navigate the data effectively. Some students pointed out wanting to learn how to download data directly from the TCGA database. Others reported that working with real patient data made an impression on them.

**TABLE 2:** Student feedback in response to each of the steps of the TCGA module.

**Step 1 of the module:** *Introduce students to the Microsoft Excel file containing data subset of interest.*
**Student feedback**
Spreadsheet with TCGA data made it clear how large the pool of genome data from cancer patients can be and how these data can be used to determine relationships between mutations and clinical patient outcomes.
Humbling to think about the data on the Excel spreadsheet coming from actual patients, some who died, and some who recovered and were able to continue living cancer-free
While spreadsheet was well organized, it took some time and exploring to understand and get a feel for the information in it.
I finally understood what it means for a patient to have “triple negative” breast cancer at the molecular level. Seeing all the potential options for these receptors lined up on the spreadsheet drove the point home.
I would be interested in learning how to create the spreadsheet with data entirely from scratch using information posted in TCGA.
**Step 2 of the module:** *Students complete a worksheet composed of 2* × *2 tables that measure associations between presence/absence of a mutation and categorical clinical phenotypes.*
**Student feedback**
Completing the worksheet helped with understanding information on dataset.
I learned new easy excel functions (like COUNTIF function) that will likely be useful later on in data and statistical analysis.
Completing the worksheet was time consuming and could easily be combined with the research question creation and analysis. This would have allowed me to come up with a question while the information in the data set is still fresh in my head.
I liked the worksheet because I was able to turn the data into relationships and percentages that were applicable to real human disease.
**Step 3 of the module:** *Students articulate a new association of interest to test (research question), create/complete the appropriate 2* × *2 tables, and calculate statistical significance of association.*
**Student feedback**
You always hear of the statistics of different cancers and stages, but with the data we were able to see the actual outcomes of real patients for our own research question, which made it more real than reading about it in a textbook.
This is the first time I have actually gotten to make my own experiment with clinical data from real humans.
I was overwhelmed at first by the amount of research questions that could be addressed with the data provided.
I tried testing several associations in the hopes of getting a statistically significant difference, but was not successful.

*For similar student feedback or statements, one representative comment was chosen and listed on this table.*

All in all, we find this is an effective way for students to experience clinically relevant inquiry in the classroom. This bioinformatics activity can also be expanded by having the students selecting the cancer of interest and pulling relevant data from TCGA.

## Data Availability Statement

The datasets presented in this study can be found in online repositories. The names of the repository/repositories and accession number(s) can be found in the article/Supplementary Material.

## Author Contributions

WH and VS designed the activities outlined in this manuscript. WH, NZ, MC, WD, AK, AR, MR, and VS implemented the activities and wrote and revised the manuscript. All authors accept and are in agreement with the content of the manuscript.

## Conflict of Interest

The authors declare that the research was conducted in the absence of any commercial or financial relationships that could be construed as a potential conflict of interest.

## Footnotes

1 https://www.cbioportal.org/

## Supplementary Material

Supplementary Appendix 1 TCGA breast cancer data subset sheet: Microsoft Excel file.

Supplementary Appendix 2 Instructional student data analysis worksheet: Microsoft Excel file.

Supplementary Appendix 3 Tips for sorting tumors in Microsoft Excel.

Supplementary Appendix 4 Microsoft Excel file to support statistical analysis.
