# Supplementary material for: Using The Cancer Genome Atlas as an Inquiry Tool in the Undergraduate Classroom
Source: Front Genet. 2020 Dec 16;11:573992. doi: 10.3389/fgene.2020.573992 (PMC7772960; doi:10.3389/fgene.2020.573992)
Supplement: Supplementary Appendix 3 — Tips for sorting tumors in Microsoft Excel. [file Table_3.DOCX]

**Appendix 3: Tips for Sorting Tumors in Microsoft Excel**

Here are some tips for using Excel functions that will group patient tumors (rows) according to a particular genomic or clinical characteristic (column).

**Sort & Filter Menu**

This menu can be found among the tools along the top of the Excel spreadsheet. If you select a particular column in your data and then click ‘Sort & Filter’, you can organize all of the tumors (rows) based on the contents of each cell in that column, for example, by alphabetical order. The purpose of using the ‘Sort & Filter’ menu is to organize your data so that it is easier to analyze subgroups within the dataset. For example, using this strategy, you can focus on Stage I tumors only.

**COUNTIF function**

Once you have sorted a group of tumors by stage (see above), this function will assist by counting the number of occurrences of a particular text in a selected area of the spreadsheet.

For example, if cancer stage information was shown in column A, and one wanted to count the number of Stage I tumors present in cells A2 through A20, the COUNTIF function can be used to accomplish this by typing the following text into a cell;

=COUNTIF(A2:A20, “Stage I”).
